# A Qualitative Insight into Pre-Departure Orientation Training for Aspiring Nepalese Migrant Workers

**DOI:** 10.3390/tropicalmed9070150

**Published:** 2024-07-05

**Authors:** Pramod Regmi, Nirmal Aryal, Edwin van Teijlingen, Radheshyam Krishna KC, Manish Gautam, Sanju Maharjan

**Affiliations:** 1 Faculty of Health and Social Sciences, Bournemouth University, Bournemouth BH8 8GP, UK; 2 Migration Health Division, International Organization for Migration, Tripoli P.O. Box 6748, Libya; 3 Anweshan, Lalitpur 44700, Nepal

**Keywords:** labour migration, pre-departure training, capacity building, Nepal

## Abstract

Pre-departure orientation training (PDOT) can help equip aspiring migrant workers with skills and knowledge to mitigate vulnerabilities throughout their migration journey, including health. In Nepal, PDOT has been mandatory since 2004 for migrant workers awaiting labour permits. The current PDOT programme includes country-specific information as well as health and well-being advice. However, the views of trainees and trainers on PDOT are largely unknown. This qualitative study aims to explore perceptions of migrant workers and relevant stakeholders on the content and delivery of PDOT. Six focus group discussions and six in-depth interviews were conducted with migrants, and eight interviews with key stakeholders were conducted. Thematic analysis resulted in five themes: (a) PDOT structure, accessibility, and implementation; (b) role of stakeholders in labour migration process; (c) coordination and governance; (d) curriculum development and relevance; and (e) capacity of trainers and effectiveness of training. Our findings emphasise the need for a more tailored curriculum with relevant information, education, and communication resources, possibly with input from former migrant workers. Regular updates of training topics and resources, as well as continued engagement with migrants after their employment, are essential for meeting the dynamic demands of the global employment market.

## 1. Introduction

Nepalese migrant workers contribute one-fifth of Nepal’s Gross Domestic Product (GDP), totalling NPR 1220.56 billion (approx. USD 11 billion) [1]. Between 2008 and 2022, over 4.7 million Nepalese sought international work permits, comprising a significant portion of South Asia’s workforce in six countries of the Gulf Coordination Council (GCC) and Malaysia [2]. These countries have sub-tropical and tropical climates. It is estimated that at least one million Nepalese work in India [2], where such a work permit is not required.

Migrant workers are particularly vulnerable to exploitation in the new country and are at risk of poor physical and mental health [3]. Therefore, it is important to identify their health needs at all stages of the migration journey [4,5,6,7]. While abroad, occupational risk as well as challenging living and lifestyle conditions, often exacerbated by limited access to health and social care, language, and cultural barriers, can result in social exclusion, and forced or voluntary overtime [3,5,8]. These factors increase their risk of injuries and illnesses, including tuberculosis, HIV (Human Immunodeficiency Virus) [9], COVID-19 [10], cardiac death [11], and kidney injury [12], as well as mental health problems such as depression and anxiety [13,14].

Since 2011, more than 600 Nepalese die every year while working abroad [2], and some 150 die each year due to traffic/workplace accidents [15,16]. Natural causes, cardiac arrest, and traffic accidents are the top three mortality causes, whilst anxiety, depression, tuberculosis, accidents and injuries, headaches, and suicide attempts are linked to morbidities [2]. 

The COVID-19 pandemic has highlighted the vulnerability of Nepalese migrant workers, emphasising the need to increase awareness and provide essential training on health and safety [10]. Many international agencies, including the WHO (World Health Organization) through its Action Plan 2019–2030 emphasise the promotion of the health of migrant workers [17] and IOM (International Organization for Migration)’s Strategic Plan 2024-28 [18] has promoted PDOT (pre-departure orientation training). Moreover, the Global Compact for Safe, Orderly and Regular Migration Framework [19] (p.10), states clearly that sending countries need to “promote multi-lingual, gender-responsive and evidence-based information campaigns and organise awareness-raising events and pre-departure orientation trainings…” Such education of migrant workers prior to departure ties in with the Sustainable Development Goals (SDGs): 3 on health, 8 on decent work, and 10 on reducing inequality [20].

One key requirement for labour approval is the completion of PDOT, which offers information and skills for safe living and employment abroad (see Supplementary File S1). It includes knowledge of labour contracts and laws, rights and responsibilities, as well as orientation to language and culture in destination countries [16]. PDOT aims to (a) reduce the vulnerability of migrant workers; and (b) enable them to maximise the benefits of overseas employment [15]. PDOT has been mandatory since 2004 [18], and there are 143 orientation centres registered with the Department of Foreign Employment (DoFE) which were licensed to provide a two-day PDOT designed by the Foreign Employment Board (FEB). There are major criticisms of PDOT highlighted as follows: a lack of effectiveness in improving migrant workers’ lives; it is too late in the pre-departure phase; and a low uptake as well as appropriateness and limited course content [14,16,21]. This study, therefore, aims to explore the various facets of the PDOT, and operation mode, and identify factors that contribute to, or hinder, migrants’ learning. 

## 2. Methods and Materials

### 2.1. Study Design

This qualitative study included focus group discussions (FGDs) and interviews with aspiring migrants, recently returned migrant workers, and key stakeholders. The latter comprised representatives of the Federation of Foreign Employment Orientation Association Nepal, an umbrella organisation of PDOT providers working closely with migrants, recruitment agents, and PDOT trainers. From the 143 DoFE registered orientation centres, a list of functional orientation centres in Kathmandu (n = 89) was identified. These were clustered into eight geographical blocks. From these blocks, 11 centres were purposively selected for FGDs and interviews with aspiring migrants. The selected orientation sites were active in offering training regularly and had a good flow of trainees. We liaised with migrant worker-related non-governmental organisations (NGOs) to help identify potential participants who had previous migration experience abroad for work (henceforth, ‘returnee migrants’). We purposively recruited participants from different age groups, ethnicities, jobs, and destination countries to capture diverse views. Inclusion criteria were (i) for aspiring migrants: those currently participating in the PDOT programme, (ii) for returnee migrants: with at least two years of experience working abroad and returned no more than two years before, (iii) for key stakeholders: people from government and NGOs working to improve labour migration and well-being of Nepalese migrants. 

FGDs with aspiring migrants were conducted in Nepali at pre-departure orientation centres, whereas interviews with separate aspiring and returnee migrants and key stakeholders were carried out at a mutually agreed location. Each FGD had between 4 and 12 individuals. Same-sex researchers (e.g., female researchers for female participants) conducted the FGDs and interviews. For each FGD, there was one facilitator and one note taker from our team. An interview guide was developed in consultation with Nepalese experts in the migration field. This guide was piloted [22] with two FGDs with migrants and one interview with a trainer. FGDs lasted 60–90 min and interviews took around 45–60 min. Interviews and FGDs were audio-recorded with the permission of our participants. All FGDs/interviews were given unique identification codes and personal identification was removed to maintain anonymity. 

Our analyses followed the six steps outlined by Braun and Clarke [23]. First, the interview recordings were transcribed and translated into English. Each transcript included field notes about the context, location, nonverbal behaviours, and a reflection on the issues identified during the session. PRR and NA (Nepalese-origin authors) independently reviewed and cross-checked the transcriptions with the audio recordings. SM independently coded all, while PRR, NA, and EvT acted as second coders. Any minor discrepancies in coding were discussed with the team. A thematic approach [24] was followed for data analysis, whereby initial codes were regrouped into sub-groups and then into overarching themes. Representative quotes from the interviews and FGDs are presented to illustrate the themes. QSR NVivo V10 was used to organise the qualitative data [25].

### 2.2. Ethical Considerations

Ethical approvals were obtained from Bournemouth University (UK) and the Nepal Health Research Council. Written informed consent [26] was obtained from all research participants. An information sheet in Nepali language was provided to participants with detailed information about the study’s purpose and procedure, voluntary participation, confidentiality, potential risks and benefits, and the complaint procedure. Each orientation centre was pre-informed about the study and their collaboration was sought.

## 3. Findings

In total, 41 aspiring migrants (26 males and 15 females) participated in six FGDs, three with each gender. Most participants were planning to go to GCC countries (Table 1). Three interviewees were aspiring migrant workers, whereas three were returnee migrants. Most interviewees were males (five out of six) and aged 21–41 years old (Table 2). Most FGD participants and interviewees were in low-skilled jobs. Eight interviews were conducted with stakeholders (six males, two females), who were in leadership roles in migrant worker-related NGOs or PDOT trainers (Table 3).

Five key themes emerged: (a) PDOT structure, accessibility, and implementation; (b) role of stakeholders in labour migration process; (c) coordination and governance; (d) curriculum development and relevance; and (e) capacity of trainers and effectiveness of training. 

### 3.1. PDOT Structure, Accessibility, and Implementation

Our aspiring migrant participants were generally happy with their training. However, migrant workers had limited choices in selecting their training sites. Most training takes place in the capital, Kathmandu, which gives trainees extra costs (i.e., travel and accommodation), for example,

*“We must stay in a hotel. There is a lot of expenses to bear besides food and accommodation…We don’t know how much more debt we’re going to be in. It’s expensive to live here. It would have been a lot easier if the training was held in our hometown. We could’ve commuted to the training from our own houses…”*

[Female aspiring migrant, FGD]

Participants also commented that recruitment agents had decided the training venue for them:

*“The manpower (recruitment) agents themselves tell us where to take the orientation class. I think we should be able to attend the training anywhere we wish to. Regarding medical check-up as well, they send us to their own relatives, and we do not have freedom to choose the place or site on our own.”*

[Returnee migrant, IDI]

Participants had conflicting views on single-sex sessions. Some thought combined classes would be preferable, for example,

*“I think, whether they are male or female, the orientation class should be given together. For instance, don’t they put both girls and boys together at school and teach them?”*

[Returnee migrant, IDI]

However, others argued that separate training may help them better understand the relevant issues. Female participants thought they may not be able to ask personal questions in front of men, e.g.,

*“…male and female should also be separately arranged to sit to enhance better understanding without feeling awkward… to ask about sensitive issues so I think better to separate sessions for male and female”*

[Female aspiring migrant, FGD]

PDOT trainers also suggested it would be better to have separate sessions for returnee migrants and first-time migrant workers.

*“Those who have already been to foreign employment aren’t interested but those who are going for the first time, they are very much interested to learn. That’s why returnee and aspiring migrant workers should be kept separately. It would be easy for us to decide on how to teach the fresh students and the returned ones.”*

[Trainer, KII]

In terms of physical space and audio-visual aids, many training centres lacked well-equipped classrooms. Participants also mentioned discomfort due to temperature and humidity in the room.

*“There are sufficient chairs at the moment, but if the number of participants increase, I don’t think they feel comfortable. It will be very congested”*

[Female aspiring migrant, FGD]

The training was in Nepali; however, the need for sessions in languages other than Nepali seemed important to some, as one trainer argued:

*“Yes, this (language barrier) thing also needs to be addressed. Some people from eastern region …are comfortable using Maithili and Hindi but less comfortable in Nepali. And some… speaking Tamang language only and they don’t feel very comfortable in Nepali. So, in such situation, it becomes quite difficult for us to teach, you see.”*

[Trainer, KII]

A female participant highlighted language issues:

*“…I don’t understand Nepali words fully and sometimes they use pure Nepali words and sentences in the videos which makes it harder for me to understand the purpose of the videos. It’s not that I don’t understand, I do but sometimes it causes difficulties in understanding.”*

[Female aspiring migrant, FGD]

Participants highlighted the importance of having study materials and expressed preference for hard copies shared during the training. They believed that such tangible resources would be beneficial for future reference.

*“They didn’t give any pamphlets or books after training. They told to buy, but I didn’t buy…”*

[Returnee migrant, IDI]

However, the federation representative, responsible for training materials and methods, presented a contrasting view, namely resource constraints, leading to certain limitations.

*“Well, you might see a shortcoming here as well. I would not like to say that. We had prepared some materials like pamphlets from our side as well to provide to them. But it has been a long time that we haven’t discussed about that. The FEB has published a book including the information that needs to be provided to them. But then, the candidates are not allowed to take the book with them.”*

[Federation representative, KII]

The FGD participants had different views regarding methods of delivery and suggested that the methods used were not up to scratch. They mentioned two options: (a) elaborate lectures; and (b) audio-visual content in multiple languages. The current information was seen as too brief, and hence, did not address their needs.

*“…would be great if they gave us detailed information about health because it was shown on the TV briefly only. Though they did tell us about HIV…through a Nepali drama which we understood well, other topics weren’t elaborated on. It was just a heading and some footage on when to wear gloves etc. It wasn’t gone through in detail. So, I think it would have been better if they elaborated more on health through lectures.”*

[Male aspiring migrant, FGD]

The trainers thought that the current training is too short to cover all aspects, and that extending it would allow more time for sharing more details, for example,

*“It takes time to explain to a group of people. We segregate specific timing (minutes) for a specific topic. But what happens sometimes is that discussion about one single topic might extend up to half an hour. So due to that reason, it would be better if the timing could be extended.”*

[Trainer, KII]

FGD participants added that training would have been better if it was spread out over one week with shorter sessions each day, to help them retain new information:

*“…better if the training duration can span for a week. Because when they teach for 5 h or 3 h continuously, we cannot memorize everything. So, I think it would have been better with less contents but for more days.”*

[Male aspiring migrant, FGD]

One NGO representative explained its agency’s support mechanism through its centres could provide more help to aspiring migrants:

*“We have over 153 Migrant Resource Centres (MRCs) operating at the municipality level. Our responsibilities involve addressing all destination-related issues. In our team, we have Outreach Coordinators. They provide regular updates. The MRCs also coordinate with us regarding cases, and people with direct access often approach us with their concerns.”*

[NGO representative, KII]

### 3.2. Role of Stakeholders in Labour Migration Process

Key informants shared the absence of regular government monitoring and evaluation of PDOT. A trainer stated that

*“There is no evaluation of the training done so far by any institutions as such… [aspiring migrants] are provided with certificates and we discuss with them any confusions or questions they are willing to ask. We ask a few of the returnee migrants to share their experiences, that’s all. No evaluation or supervision-related activities are undertaken.”*

[KII, Trainer]

All training institutes in this study had the biometric system in place, which meant migrants’ participation in training could easily be verified at the airport. However, some key informants mentioned the additional benefit for the DoFE and FEB in monitoring the implementation of PDOT:

*“The participants in the training are to be registered through biometrics into the system, so if foreign employment board is willing enough to supervise and monitor the activities conducted by each training centres, then they [Board] can easily execute it, and get information on how many migrants are trained from each institute. This could aid in monitoring mechanism.”*

[NGO representative, KII]

All female trainees must pay NRs 700 rupees (about USD 5) for enrolment, which should be reimbursed later. But the estimate from one stakeholder is that barely ten percent of women go through the cumbersome and expensive process of trying to get reimbursed:

*“…money would be returned to them only after all their procedures are done and their documents are completed. It would be such a hassle for them to go find the board… That person would already have spent NRs. 1000 [US$ 7.50] so why would they take to trouble to get NRs. 700 [US$ 5] back. So, it is not practical at all.”*

[Federation representative, KII]

### 3.3. Coordination and Governance

The training does not make clear what the various organisations, both governmental and private, contribute to the welfare of migrant workers, nor how this sector is coordinated. One participant commented on this confusion:

*“One main problem is that there is no …NGOs or any other organization who would support us in this field [quality assurance of migration process]. I don’t know why they [other organizations] don’t, whether they perceive us [Federation] negatively or is it that they don’t consider as capable, I don’t know what exactly it is. But no organization has collaborated with us to work. We have the plan as well as the program. So, when we have both, we could collaborate and work together. But they don’t have any concern upon it.”*

[Federation representative, KII]

Whilst NGO representatives reported that migrant workers trust recruitment agents more than NGOs and training providers. These agents serve as the primary source of information and aspiring migrants depend on their information when making important decisions in their lives.

*“They trust [recruitment agents], and if they provide authentic information, they won’t face any difficulty. It is easier now as there are three levels of government.”*

[NGO representative, KII]

Most stakeholders from a range of institutions mentioned that better monitoring by all levels of government and regulatory bodies is needed to ensure the PDOTs offer quality training. Currently, recruitment agencies refer migrant workers to training centres, but there is no verification of attendance for centres without the biometric system, so there is no way of knowing if prospective migrant workers attended. Most thought the government did not have a monitoring and evaluation system in place.

*“…recruitment agency drops the migrant workers in the training centre. It should be the government’s responsibility to make sure that the aspiring migrants have [=participated].”*

[NGO representative, KII]

The need for more involvement of local governments with better communication and coordination was emphasised by many. Some felt that local governments should collaborate with training centres and recruitment agencies to gather accurate data on aspiring migrant workers and store essential information about migrants for the future.

*“…it should be initiated by the local level government. Depending on the number of aspiring migrants, nearby rural municipalities should coordinate with one another and open a common training centre.”*

[Trainer, KII]

### 3.4. Curriculum Development and Its Relevancy

Though the PDOT curriculum was revised in 2021, the full curriculum is not in the public domain, nor have training centres been given a date for its official start. Most trainers in our study argued that its design and development had not included trainers, the sole implementors of the programme.

*“I am a board member, yet I am unaware of curriculum development… Trainers’ experiences and knowledge is an important aspect for curriculum development.”*

[Trainer, KII]

The current content covers fundamental topics such as occupational safety, alcohol, and cigarette consumption, HIV and STI (sexually transmitted infections), insurance, traffic rules, and legal norms of the host country. Most participants perceived this content as helpful, however, a few FGD participants noted that content is more useful for new migrant workers than for returnees.

*“Others who didn’t know* (i.e., those who are travelling for the first time) *got to understand about all these too. So, I think it is good.”*

[Male aspiring migrant, FGD]

Many FGD participants feel that the PDOT has limitations in preparing migrant workers for the experiences and challenges abroad, as nothing can prepare one better for real life than the actual experience:

*“We will have to go through whatever it is only after going there. It is the matter of experience there which the orientation class cannot provide.”*

[Returnee migrant, IDI]

The content is still not good enough, with a gap in country-specific content:

*“It is really important to know about the country that you are going. The training should be country-specific and country-focused.”*

[Aspiring migrant, FGD]

FGD participants claimed they were informed about workplace safety precautions, self-care tips before, during and after work, and maintaining a healthy lifestyle to work efficiently. Overall, the FGD participants found the occupational safety-related contents to be informative and useful:

*“We now know that there are different risks in different jobs, and we ourselves have to follow safety precautions.”*

[Aspiring migrant, FGD]

On the contrary, views on the inclusion of sensitive topics, such as sexual health, were dependent on the number of females present in the training session. Remarkably, when there were more female aspiring migrant workers in training sessions, topics like STIs and HIVs were discussed. The practice of omitting these issues when there were only men may have a negative impact on their knowledge and behaviour:

*“Well, when only males are present in the class, the sensitive issues like you said (sexually transmitted diseases and HIV/AIDS) are discussed less. But if the females are also present, we have to include all those sensitive issues in our discussion.”*

[Trainer, KII]

There is a session on Nepal’s government’s policies and right to employment, provisions related to foreign employment, and the role of local government. Participants claim such topics only lengthened the training and distracted from the main goal of the orientation programme.

*“… there is no necessity for us to teach about Nepal’s constitution to the people going for foreign employment. This is only included to stretch the time and make it lengthy. They are trying to increase the timing of the training, but they aren’t able to explain the gist of the subject.”*

[Federation representative, KII]

Additionally, some also expressed dissatisfaction with the information covered, recommending that topics such as self-defence and sexual harassment should be included:

*“It [sexual harassment-related content] wasn’t gone through in detail. So, I think it would have been better if they elaborated more on health-related through lectures and any other available methods. I think it would be helpful if they taught us that by acting or showing us self-defence instead of telling us what to do. For example, what to do, how to dodge if they grab our hair etc, those type of classes.”*

[Female aspiring migrant, FGD]

Additionally, the FGD participants acknowledged the importance of mental health and well-being in destination countries since they live a monotonous life within the limited circle of close colleagues. Their social and work environment could exacerbate work or financial stress, and homesickness. In the following quote, this female participant interestingly refers to her employer as the ‘owner’:

*“That is our worries and woes. Sometimes we might not be able to send money home and sometimes our owners might not be happy with our work. That affects our mind.”*

[Female aspiring migrant, FGD]

Though the trainers claim that an hour of session is dedicated to mental health and well-being, trainees indicated that the topic was merely mentioned, but not explained in any detail.

*“[Trainer] earlier mentioned depression. He asked if we were familiar with the terms and condition of health. [Trainer] told us we should not take any tension when we go there, he had advised that. But how can it be addressed? They asked something like that. Those things were discussed but they did not explain it in detail.”*

[Male aspiring migrants, FGD]

### 3.5. Capacity of Trainers and Effectiveness of Training

Our findings indicate that neither the federation staff nor the training institutes were aware of the selection criteria and current process of Training of Trainers (ToT). The criteria for selecting trainers are unclear on the web:

*“Before when we started to run the training centre, there was a criterion that the trainer should at least have completed a master’s degree. That accounted for a certain percentage. And the other criteria for the trainer were that they should have completed a bachelor’s degree and have some experience in foreign employment. But now both the criteria are not there, and they haven’t said anything regarding the trainers. Now, anyone who has completed their +2 level can become a trainer.”*

[Federation representative, KII]

As reported in one interview, the latest refresher training was years ago:

*“We had conducted the refresher training in 2068 (=2011/12) on behalf of the board [FEB]. Then they produced new ToTs in 2070 (2013/2014) and then in 2073 (2016/17).”*

[Federation representative, KII]

Trainers in our study shared how the lack of capacity building hampered trainers’ ability to deliver information effectively.

*“We lack adequate training and resources. We teach migrants based on our limited capabilities, unable to adequately cover all necessary information. We ourselves would benefit from further capacity building and training to enhance our knowledge and teaching skills.”*

[Trainer, KII]

Another trainer added

*“I attended Training of Trainers about 13 years ago…I have been giving training to migrant workers since then, without any refresher training…”*

[Trainer, KII]

It is also evident from our findings that trainers are being hired on an ad hoc basis without any proper regulations regarding their hiring terms and expertise. Many key stakeholders questioned the quality of the service provided. Currently, one female trainer is mandatory for each training centre to address the needs of female trainees; however, not all centres have female trainers and trainees.

*“Well, it has been clearly mentioned in the curriculum …in every institution, there should be a female trainer for the female candidates to take an hour’s class, but it is not practically implemented…”*

[Federation representative, KII]

## 4. Discussion

The present study highlights current inadequacies in the PDOT curriculum, its delivery, and impact, especially on health-related components. The poor running of PDOT is exemplified by the lack of surveillance mechanisms for training centres and coordination. Notably, the PDOT curriculum has limited or no coverage of topics such as self-defence, sexual harassment, mental health, sudden deaths, cardiometabolic, and kidney risks, which are of the utmost importance in destination countries [27,28].

Some gaps in the curriculum may be attributable to a lack of input in its development from trainers, recruitment agents, migrant employers, and migrants. We suggest that the government involve these stakeholders in further policy-making and curriculum development processes. The vulnerability of migrants frequently originates from their source communities, where decisions to migrate are frequently influenced by inaccurate information and a strong urge to leave. In addition to having unrealistic expectations, many migrants lack essential knowledge about health, safety, and infectious diseases [29,30]. Unfortunately, Public Health agencies have not been able to push the needs of migrant workers higher up the government’s agenda. Studies suggest shorter pre-departure orientation sessions insufficiently address migration risks, rights, and social norms in host countries [29]. Overall, the pre-departure session was not aligned with actual migration trends and requirements, highlighting the necessity of a context-specific and evidence-based approach [31]. Effective PDOT for South Asian workers, not just those from Nepal, requires a comprehension of regional trends and larger social influences.

In addition, the qualifications of PDOT trainers and the quality of training provided are pressing concerns. Inconsistencies exist due to ambiguity around appointments and qualifications of trainers, something which a better targeted policy on training could overcome. Without regular refresher training, trainers’ competence and knowledge may deteriorate. Moreover, there is a need for multilingual trainers to cater for Nepal’s diverse linguistic landscape.

Usually, aspiring migrants congregate in Kathmandu as Nepal really has only one large functional international airport; migrants also need to come to the capital for sorting out documentation (work permits, visas, etc.) and PDOT, which puts substantial additional costs on migrants. It also means that most migration information and support is confined to urban centres. Through community-based information programmes and capacity-building initiatives, NGOs attempt to reduce knowledge gaps in rural areas, for example, in Indonesia [15]. Likewise, the content delivered methods of orientation delivery patterns and its relevance to the context in PDOT played a crucial role among aspiring migrants. We suggest that former migrant workers might be trained to contribute to the more practical aspects of PDOT [32,33]. Additionally, the length of training, enrolment processes, and physical facilities in orientation centres had a significant impact on the PDOT.

Key stakeholders agree that there is an urgent need to increase the accessibility of migration information, as was also outlined in the Global Compact for Migration [19] and advocated in the WHO Action Plan for Refugees and Migrants [17]. We suggest there could be a role here for major development partners, including the ILO, the IOM (International Organization for Migration), the WHO, and NRNA (Non-Resident Nepalese Association), bringing an international perspective. The Government of Nepal may want to consider incorporating a global input into its policy-making processes around migration, not just those related to Public Health. This could improve the decision-making of prospective migrants, potentially reducing their reliance on brokers/recruitment agencies, guiding them toward secure migration channels, and raising awareness of their rights and responsibilities [15]. It is important to have collaborative efforts that foster greater coordination between public and private entities, as well as training institutions, as part of a viable strategy for maximizing the use of available resources on migration and migrants’ health. In 2010, the IOM supported the opening of the MRC in Kathmandu. Its primary mission was to provide information on secure migration via counselling mechanisms such as face-to-face meetings, email correspondence, and telephone consultations. In collaboration with the migrant workers’ NGO *Pravasi Nepali Coordination Committee* (PNCC), MRC expanded its scope by establishing foreign employment centres in several districts and providing, among other services, pre-departure orientation. Establishing a system for periodic updating, reproduction, and distribution of these materials could offer a targeted approach to PDOT [15].

This study highlights the increasing demand for country-specific, skill-specific, and gender-specific interventions for potential migrants; a similar insight was reported in Tajikistan [33]. Given the growing range of destination countries and migrant occupations, this might pose a challenge. Although a large proportion of migrant workers still travel to a limited number of countries, the first step could be the compilation of an inventory of customised materials produced by government agencies, and NGOs. The latter should also be invited to help improve PDOT.

Key limitations of this study are that because this qualitative study was by its nature small scale and with purposive sampling, we may have missed other voices. Only a few participants were returnee migrants as most were aspiring migrant workers. Also, our participants represented PDOT centres in Kathmandu, whilst most training centres (91 of 143) are in the capital; incorporating views of participants outside Kathmandu could have provided different views. Also, most participants are males (not surprising as more than 90% of Nepalese labour migrants are males), thus findings related to gender should be cautiously interpreted.

Our study generated the following recommendations which are grouped by targeted recipients: (1) the Government of Nepal; (2) PDOT training organisations; (3) migrants themselves; and (4) researchers. First, government agencies need to better monitor training organisations. Second, governments and training organisations need to update their curricula involving all stakeholders to incorporate new emerging health issues. Third, training organisations should allow sufficient time to equip would-be migrants with PDOT. Fourth, training organisations should improve the classroom environment. Fifth, training organisations need to ensure proper education of their trainers and regular refresher courses. Sixth, the government and training agencies should work together to create more PDOT centres in other parts of the country. Seventh, former migrant workers should be involved in the delivery of the PDOT. Eighth, MRCs should be better funded to provide relevant and up-to-date information. Ninth, all stakeholders need to be aware of the growing number of countries where Nepalese migrant workers go; each new country requires a new set of information and guidance. Tenth, we recommend conducting a study focusing on the particular training needs of migrant women. Finally, we need a well-funded longitudinal study of would-be migrants to assess the effectiveness of PDOT using a before-and-after study for a range of different people and destination countries.

## 5. Conclusions

Nepal’s PDOT framework provides a structured approach to equip and protect its labour migrants. However, there are manifested gaps, most notably in curriculum design, geographical accessibility, stakeholder collaboration, and trainers’ proficiencies. The government should be proactive in revising the curriculum periodically with time and context-relevant content and render more efforts in monitoring and evaluating PDOT. Our findings emphasise the need for a comprehensive surveillance system and a more tailored curriculum. Regular updating of training materials and post-employment engagement with migrants are essential in refining the curriculum and meeting the dynamic demands of the international employment landscape; this process should be supported by international agencies working in the field. Strengthening and implementing policies more effectively could facilitate federal and local governments’ ability to collaborate with training centres, and hence, create locally accessible PDOT to all prospective migrant workers. This synthesis of findings emphasises the necessity for immediate actions to bridge these gaps and ensure the sustained welfare of Nepalese migrant workers. Moreover, since little international literature exists, it is advisable to conduct a cross-national comparative study incorporating Asian countries with a large number and/or proportion of migrant workers abroad, e.g., countries such as the Philippines, Bangladesh, and Indonesia.

## Figures and Tables

**Table 1:** Characteristics of FGD participants (all aspiring migrants).

FGD	Age Range (Years)	Gender	Ethnicity	Destination	Occupation Abroad
1	21–33	All female	Janajaati—1 Chhetri—1 Brahmin—1 Dalit—1	Maldives Qatar Oman—2	Housework Cleaner Babysitter Housework
2	23–36	All female	Dalit—2 Chhetri—3 Janajaati—2	UAE—7	Cleaner—6 Sales—1
3	24–40	All female	Janajati—1 Chhettri—1 Dalit—1 Brahmin—1	Qatar—2 Dubai—2	Waitress—1 Cleaner—2 Labour—1
4	23–37	All male	Chhetri—2 Janajaati—6 Dalit—1	Saudi Arabia—6 Maldives—1 Qatar—1 UAE—1	Scaffolding—4 Helper—2 Cook—1 Driver—1 Cleaner—1
5	20–38	All male	Chhettri—2 Janajati—1 Dalit—2	Saudi Arabia—2 UAE—3 Malaysia—1	Helper—3 Labour—1 Security guard—1 Cleaner—1
6	21–45	All male	Janajati—1 Chhettri—9 Dalit—2	Romania—10 Kuwait—1 Malaysia—1	Cook—1 Labour—4 Carpenter—5 Security guard—1 Conductor—1

**Table 2:** Characteristics of in-depth interview participants.

Interview	Age (Years)	Gender	Ethnicity	Destination Countries	Occupation Abroad	Type of Migrant
IDI 1	21	Male	Brahmin	UAE	Helper	Aspiring
IDI 2	41	Male	Terai Chettri	UAE	Cleaner	Aspiring
IDI 3	22	Female	Janajaati	Romania	Cleaner	Aspiring
IDI 4	29	Male	Janajaati	Malaysia	Industrial work	Returnee
IDI 5	34	Male	Hill Dalit	Malaysia	Security worker	Returnee
IDI 6	32	Male	Madhesi Dalit	Qatar	Cleaner/Painter	Returnee

**Table 3:** Characteristics of KII participants.

KII	Participants Role	Gender
KII 1	Chairperson	Male
KII 2	Chairperson	Male
KII 3	Programme Officer	Male
KII 4	Managing Director and Trainer	Female
KII 5	Trainer	Female
KII 6	Trainer	Male
KII 7	Trainer	Male
KII 8	Trainer	Male

## Data Availability

The data presented in this study are available on request from the corresponding author.

## Supplementary Materials

The following supporting information can be downloaded at: https://www.mdpi.com/article/10.3390/tropicalmed9070150/s1, Supplement File S1. PDOT contents.
