# Supplementary material for: A Qualitative Insight into Pre-Departure Orientation Training for Aspiring Nepalese Migrant Workers
Source: Trop Med Infect Dis. 2024 Jul 5;9(7):150. doi: 10.3390/tropicalmed9070150 (PMC11281639; doi:10.3390/tropicalmed9070150)
Supplement: Supplementary file 1 [file tropicalmed-09-00150-s001.zip › tropicalmed-3017436-supplementary.docx]

**Supplement file S1*.*** PDOT contents*.*

| **Subject/Duration** | **Sections** | **Sub-sections** |
| --- | --- | --- |
| **1.** Things to understand about Nepal's constitution and laws related to foreign employment) [45 Minutes] | **Section 1:** Constitution of Nepal (right to employment, provisions related to foreign employment and role of local government) |  |
|  | **Section 2**: Safe Foreign Employment Provisions as per the Foreign Employment Act, 2064 and Regulations, 2064 |  |
| **2**. Easy and safe journeys to destination country [90 Minutes] | **Section 1**: Information and preparation in advance for easy and safe travel to the destination country) | Information about materials and documents to be brought |
|  |  | Documents to be left with the family |
|  | **Section 2:** Travel information from point of departure to point of arrival) | Domestic airport usage and procedures |
|  |  | Things to consider when travelling by plane |
|  |  | Procedure to be followed in case of transit |
|  |  | Matters to be considered before reaching immigration after landing the plane |
|  |  | immigration procedures to be followed in foreign immigration |
|  |  | Procedures to be followed after exiting a foreign airport |
| **3.** Information about contracts, personal conduct, and stress management) [60 Min] | Subjects included in the contract |  |
|  | Conduct and behavior to be followed |  |
|  | Positive thinking and behavior |  |
|  | Identifying and managing stress |  |
| **4.** Information about precautions to be taken at the workplace and safety from possible accidents  [60 Min] | Discipline and conduct to be followed in the workplace |  |
|  | Causes and prevention of workplace accidents |  |
|  | Risks that may arise from the use and non-compliance of protective equipment |  |
|  | Emergency Precautions |  |
|  | The main points to be considered while using the equipment according to the nature of the work |  |
| **5.** Basic information about health and occupational safety [150 Min] | **Section 1:** Basic information about health protection | Personal health, safety, and prevention |
|  |  | Pre-departure health check-up |
|  |  | Communicable and non-communicable diseases and prevention |
|  |  | Basic information about sexual and reproductive health protection |
|  |  | Violence and harassment |
|  | **Section 2:** Occupational health protection | Things to know about your health and safety in the workplace |
|  |  | Health problems that may occur according to the nature of the workplace and how to avoid them |
|  |  | Work-related diseases |
| **6.** Provisions on Wage Management [30 Min] | Information regarding utilization of remittances |  |
|  | Savings and Investments |  |
|  | Use of ATM |  |
| **7.** Information on the use of communication and information technology  [30 Min] | Proper use of telephone and mobiles |  |
|  | Careful use of social media |  |
|  | Use of different applications |  |
| **8.** Information about support agencies and how to get help [30 Min] | Constitutional commission and government agencies that prioritize cooperation |  |
|  | Government organizations that focus on cooperation |  |
|  | Procedure for getting help in case of problem |  |
